# Epidemiologic features of upper gastrointestinal tract cancers in Northeastern Iran

**DOI:** 10.1038/sj.bjc.6601737

**Published:** 2004-03-16

**Authors:** F Islami, F Kamangar, K Aghcheli, S Fahimi, S Semnani, N Taghavi, H A Marjani, S Merat, S Nasseri-Moghaddam, A Pourshams, M Nouraie, M Khatibian, B Abedi, M H Brazandeh, R Ghaziani, M Sotoudeh, S M Dawsey, C C Abnet, P R Taylor, R Malekzadeh

**Affiliations:** 1 Digestive Disease Research Center, Tehran University of Medical Sciences, Shariati Hospital, North Kargar Avenue, Tehran, Iran; 2 Cancer Prevention Studies Branch, National Cancer Institute, Bethesda, MD, USA; 3 Golestan University of Medical Sciences, Gorgan, Iran

**Keywords:** oesophageal cancer, gastric cancer, Turkmen, Iran

## Abstract

Previous studies have shown that oesophageal and gastric cancers are the most common causes of cancer death in the Golestan Province, Iran. In 2001, we established Atrak Clinic, a referral clinic for gastrointestinal (GI) diseases in Gonbad, the major city of eastern Golestan, which has permitted, for the first time in this region, endoscopic localisation and histologic examination of upper GI cancers. Among the initial 682 patients seen at Atrak Clinic, 370 were confirmed histologically to have cancer, including 223 (60%) oesophageal squamous cell cancers (ESCC), 22 (6%) oesophageal adenocarcinomas (EAC), 58 (16%) gastric cardia adenocarcinomas (GCA), and 58 (16%) gastric noncardia adenocarcinomas. The proportional occurrence of these four main site-cell type subdivisions of upper GI cancers in Golestan is similar to that seen in Linxian, China, another area of high ESCC incidence, and is markedly different from the current proportions in many Western countries. Questioning of patients about exposure to some known and suspected risk factors for squamous cell oesophageal cancer confirmed a negligible history of consumption of alcohol, little use of cigarettes or nass (tobacco, lime and ash), and a low intake of opium, suggesting that the high rates of ESCC seen in northeastern Iran must have other important risk factors that remain speculative or unknown. Further studies are needed to define more precisely the patterns of upper GI cancer incidence, to test other previously suspected risk factors, and to find new significant risk factors in this high-risk area.

A cancer registry maintained by the International Agency for Research on Cancer (IARC) and the Institute of Public Health Research of Tehran University (IPHR) showed that from June 1968 to June 1971 the age-adjusted incidence rates of oesophageal cancer (EC) for both men and women from Golestan Province who lived in the steppe grasslands of the Turkmen plain and neighbouring hills in the north-eastern part of the province were higher than 100/100 000 and thus among the highest rates in the world (Kmet and Mahboubi, 1972; Mahboubi *et al*, 1973). Moreover, whereas in Western countries EC has been mainly a disease of men who smoke and drink alcohol heavily (Munoz and Day, 1996; Brown *et al*, 2001), in the very high incidence areas of Golestan Province (inhabited almost entirely by Turkmens) there were very low rates of smoking and drinking and EC was slightly more common in women than in men. The cancer registry also showed sharp gradients of incidence over relatively short geographical distances. Rates fell westward for some 300 km along the southern Caspian littoral (a densely populated area inhabited largely by Persians) through Mazandaran Province and into Gilan Province (a much wetter region of rice paddies and orange groves) where the incidence was lower than 10 per 100 000 in women and between 10 and 20 per 100 000 for men.

Ecological and nutritional studies conducted by IARC and IPHR to establish the epidemiologic features and to investigate the aetiology of EC throughout the Caspian Littoral including Golestan Province (Joint Iran-IARC Cancer Study Group, 1977) found geographical associations of incidence with a number of variables. A subsequent case–control study investigated these associations further and established some risk factors, especially poverty and a restricted diet very low in fresh fruit and vegetables (Cook-Mozaffari *et al*, 1979), factors that have since been shown to be associated with an elevated risk for oesophageal cancer in almost all countries where diet has been studied (Munoz and Day, 1996), but it found no clear evidence for other potential risk factors such as hot tea or nass consumption. However, the studies in Iran were discontinued due to the sociopolitical changes there in 1979, before the complete patterns of incidence and the full complement of risk factor results could be established. Some of the major questions that were left unanswered were the precise incidence of EC and GC and the proportions of different histologic subtypes of oesophageal tumours in the different ethnicities in Golestan Province, partly because population data by ethnic group were not available and partly because the lack of endoscopy and histological diagnoses may have led to incorrect designation of tumour location and histology for a proportion of patients. It was postulated at the time that almost all the oesophageal cancer cases in this area were of squamous origin, but 98% of the tumours were diagnosed by radiology or by clinical methods alone and only 2% had histological confirmation (Mahboubi *et al*, 1973).

In August 2001, we opened a referral clinic for upper gastrointestinal (GI) tract cancers, the Atrak Clinic, in the principal city of the region, Gonbad, at the Khatam hospital, the largest hospital of eastern Golestan. The Clinic is affiliated with and staffed by the Digestive Disease Research Centre (DDRC) of Tehran University of Medical Sciences. Our aims were both to provide care for patients and to build on the previously unfinished research studies. Here, we report on the patients seen in the first 24 months of Atrak Clinic, including the location and histology of the oesophageal and gastric cancers and some epidemiological features of these cancer cases. We also compare our findings with those of the previous studies in Iran and with the epidemiological features of GI cancers seen in Linxian, China, another area with extremely high rates of EC.

## MATERIALS AND METHODS

The majority of cancer patients in eastern Golestan present first to the local general practitioners or to the medical and surgical specialists in the area, and only a small group of patients are first diagnosed in major cities outside the area. Before the study began, the investigators contacted all of the local medical practitioners and asked them to refer their patients with suspected GI tract cancers to the Atrak Clinic. From August 2001 to August 2003, 682 patients were referred to the Atrak Clinic. Based on the results of a recent cancer surveillance study and an ongoing cancer registration in Golestan Province, we have shown that approximately 70% of the incident cases of oesophageal cancer recorded in the eastern part of Golestan Province during the study period were referred to the Clinic (unpublished data), so the results of this report may be generalised to represent the experience of eastern Golestan Province.

All the 682 patients referred to the Atrak Clinic were suspected of having upper GI cancers. After signing an informed consent, the patients were interviewed by a physician using a structured questionnaire and underwent physical examination followed by oesophago-gastro-duodenal videoendoscopy. Intravenous Midazolam (5 mg) and 10% lidocaine spray to the pharynx were used as premedication. Local medical specialists, who had been given specific training, performed the endoscopies using Olympus GIF-XQ230 and Pentax EG-2900 video endoscopes. At least four biopsies were obtained from all of the tumours that were found during endoscopy and standard biopsies were taken from the antrum, the gastric body (lesser curvature), the cardia and the oesophagus in all patients. Two more biopsies were taken from columnar-lined distal oesophagus, if such tissue existed. The endoscopic data were entered on predesigned forms, and the location of the tumours was either captured and registered electronically (90% of the tumours), or precisely drawn on a specially designed form. An experienced endoscopist (R Malekzadeh) reviewed both the endoscopic reports and the captured images to confirm the exact site of the tumours. Biopsy specimens were oriented and spread on strips of filter paper and fixed immediately in 10% buffered formalin. The samples were sent to the DDRC, in Tehran, where they were embedded, sectioned and stained with haematoxylin and eosin and examined by experienced DDRC pathologists (M Sotoudeh and B Abedi).

The cancers were classified into four groups: oesophageal squamous cell carcinoma (ESCC), oesophageal adenocarcinoma (EAC), gastric cardia adenocarcinoma (GCA) and gastric noncardia adenocarcinoma (GNCA). Adenocarcinomas of the stomach were classified as intestinal or diffuse type using Lauren's classification criteria (Lauren, 1965). Gastric cardia tumours were defined as adenocarcinoma with an estimated point of origin within 1 cm proximal or 3 cm distal of the oesophago-gastric junction.

The study was reviewed and approved by the Institutional Review Boards of the DDRC and the US National Cancer Institute.

## RESULTS

Among the initial 682 patients, 398 were suspected at endoscopy of having upper GI tract cancer and 370 subsequently had histological confirmation of malignancy. Table 1

**Table 1:** Site and histological diagnosis of the upper gastrointestinal cancers in patients referred to the Atrak Clinic

**Histological type**	**Frequency**
Oesophageal squamous cell carcinoma	223 (60%)
	
*Oesophageal adenocarcinoma*	22 (6%)
Intestinal type	11
Diffuse type	8
Not determined	3
	
*Gastric cardia adenocarcinoma*	58 (16%)
Intestinal type	35
Diffuse type	16
Not determined	7
	
*Gastric noncardia adenocarcinoma*	58 (16%)
Intestinal type	34
Diffuse type	17
Not determined	7
	
*Others*	9 (2%)
Oesophageal adenosquamous carcinoma	4
Gastric lymphoma	1
Duodenal adenocarcinoma	4

summarises the sites and the histological diagnoses found in these 370 patients. ESCC was the most common type of cancer seen at the Atrak Clinic, constituting 91% of all oesophageal cancers and 60% of all upper GI cancers. The middle third of the oesophagus was the most frequent site of origin of these tumours: 32 (13%) of all oesophageal tumours were found in the upper third, 135 (54%) in the middle third, and 82 (33%) in the lower third. EAC was relatively uncommon, comprising only 9% of all oesophageal cancers and 6% of all upper GI cancers. The gastric cancers were evenly split between GCA and GNCA. Among patients diagnosed with gastric adenocarcinoma, the intestinal type of cancer was approximately twice as frequent as the diffuse type.

Table 2

**Table 2:** Demographic characteristics of cancer and noncancer patients referred to the Atrak Clinic

	**Oesophageal cancer**		**Gastric cancer**		**Patients with no endoscopically suspicious lesion (*n*=284)**
**Characteristic**	**Squamous cell carcinoma (*n*=223)**	**Adenocarcinoma (*n*=22)**	**Cardia (*n*=58)**	**Noncardia (*n*=58)**	
Median age (interquartile range)	65 (55–72)	66 (58–74)	66 (57–73)	64 (59–72)	60 (47–69)
Sex (male)	116 (52%)	13 (59%)	42 (72%)	44 (76%)	154 (54%)
Residence (urban)	70 (31%)	7 (32%)	17 (29%)	23 (40%)	129 (45%)
Ethnicity (Turkmen)	119 (53%)	14 (64%)	31 (53%)	21 (36%)	92 (32%)

summarises the demographic characteristics of the 361 cancer patients diagnosed with ESCC, EAC, GCA or GNCA, and of the 284 patients with no endoscopically suspicious lesion (noncancer patients). Median age was close to 65 years in cancer patients and 60 in the noncancer patients. The male to female ratio was close to 1 : 1 in ESCC, EAC patients and noncancer patients and close to 3 : 1 in GCA and GNCA patients. In all, 31% of the ESCC, EAC, and GCA patients, 40% of the GNCA patients and 45% of the noncancer patients were from urban areas. There were no significant differences in the proportion of urban residence among the first three cancer groups. A higher proportion of ESCC, EAC and GCA, but not GNCA patients, were from rural areas compared with the noncancer patients (*χ*^2^ 12.9, *P*<0.001 and 0.65, *P*=0.42, respectively). Totally, 54% of ESCC, EAC and GCA patients, but only 36% of GNCA and 32% of noncancer patients were Turkmens; the proportions of Turkmens were significantly higher for both oesophageal and GCA patients, but not for GNCA patients, than for noncancer patients (*χ*^2^ 28.2 *P*<0.001 and 0.32 *P*=0.57). There were no significant differences in Turkmen ethnicity among the ESCC, EAC and GCA groups (*χ*^2^ 0.86 and *P*=0.66).

Table 3

**Table 3:** Prevalence of some suggested oesophageal cancer risk factors in patients referred to the Atrak Clinic

	**Oesophageal cancer**		**Gastric cancer**		**Patients with no endoscopically suspicious lesion (*n*=260)**
**Characteristic**	**Squamous cell carcinoma (*n*=144)**	**Adenocarcinoma (*n*=12)**	**Cardia (*n*=42)**	**Noncardia (*n*=40)**	
Alcohol (ever consumed)	1 (1%)	1 (10%)	0 (0%)	0 (0%)	5 (2%)
Smoking (ever smoked)	39 (27%)	3 (25%)	14 (33%)	12 (30%)	51 (20%)
Nass (ever used)	18 (12%)	3 (25%)	6 (14%)	6 (15%)	23 (9%)
Opium^a^					
New users	9 (6%)	0 (0%)	4 (10%)	6 (15%)	15 (6%)
Old users	39 (27%)	4 (33%)	11 (26%)	14 (35%)	56 (22%)
Any risk factor^b^	69 (48%)	5 (42%)	23 (55%)	22 (55%)	102 (39%)

a New user refers to those who started opium use during the year before the diagnosis was made. Old user refers to those started opium use earlier than 1 year before the diagnosis.

b Any risk factor refers to using at least one of the four risk factors (alcohol, cigarette, nass or opium).

shows the prevalence of previous exposure to some known or suspected risk factors for ESCC, including alcohol consumption, cigarette smoking, the chewing of nass and opium consumption. These data were available for only 238 cancer patients and 260 noncancer patients. Only two (1%) of the cancer patients and five (2%) of the noncancer patients had ever consumed alcohol. In total, 29% of all cancer patients and 20% of noncancer patients had ever smoked. Smoking was more frequent among men: 39% of all male cancer patients and 12% of all female cancer patients had ever smoked, with rates being very similar across the cancer groups. Nass was used by 14% of cancer patients and 9% of noncancer patients, and opium consumption was seen in 37% of all cancer patients and 29% of noncancer patients. To investigate whether opium consumption was an old habit or it was used as a medication to alleviate pain from a recent disease, we asked the patients about the time they had started using opium. Those who had started in the year prior to being referred to Atrak Clinic were classified as new users. In all, 27% of ESCC patients had started their opium use earlier than 1 year ago, and 6% had started in the year before being referred to the Atrak Clinic. Taken together, 50% of all cancer patients and 39% of noncancer patients had ever used at least one of these risk factors.

## DISCUSSION

In total, 245 cases of oesophageal cancer and 116 cases of gastric cancer were referred to the Atrak Clinic in the first 24 months after it was opened. Assuming that the proportion of UGI cancer cases referred to the clinic was independent of tumour site, we can conclude that EC is approximately twice as common as GC in eastern Golestan Province. In addition, almost half of the GC cases occurred in the cardia of the stomach. A predominance of EC cases over GC cases and a large number of GC cases originating in the cardia are common findings in populations at high risk for EC, but are unusual findings for other areas of the world. For example in Linxian, China, another area with very high rates of EC, the EC : GC ratio is also greater than one and the GCA cases far outnumber the cases of GNCA (Blot *et al*, 1993), but in most areas of the world, the opposite proportions are found.

Compared to the cancer registry rates for 1969–1971 in this area, which showed an EC : GC ratio of 8 : 1 (Mahboubi *et al*, 1973), the current much lower 2 : 1 ratio might seem to imply that changes in incidence have occurred for either or both sites in eastern Golestan, but a substantial part of the difference may be an artefact of diagnostic method and classification. ESCC, EAC and GCA all usually present with dysphagia as the dominant symptom, while GNCA usually presents with abdominal pain without dysphagia. In the 1970s, when endoscopy was not available in the region, ESCC, EAC and GCA were probably all diagnosed as EC, while only GNCA was diagnosed as GC. Also supporting this possibility is the observation that in the 1970s more than half of the oesophageal cancers were located in the lower third (Mahboubi *et al*, 1973), while in the present series only a third of these tumours were localised to that site. This difference could be explained by some of the cancers previously classified as lower third oesophageal tumours now being more accurately classified as GCA. If one combines the current figures for ESCC, EAC and GCA, the EC : GC ratio is 5 : 1. Of course, some real changes in the incidence of these tumours may also have occurred and some of the changes may be due to differential levels of referral, but much of the apparent change in their proportions appears to be due to these changes in diagnostic method and classification.

ESCC is the dominant histological type of EC in eastern Golestan Province, since more than 90% of the EC cases in this series were ESCC. This predominance of ESCC : EAC is also seen in other high-risk EC populations, but is not as common in populations with low EC risk. In Linxian, for example, virtually all EC cases are ESCC, but in the US, the proportion of EAC cases among all oesophageal cancer cases has increased from 14 to 51% from 1975 to 1998, and intestinal type EAC is now the predominant histological type (Polednak, 2003). Similar sharp increases in incidence of EAC have also been reported from Europe (Walther *et al*, 2001; Powell *et al*, 2002), but no such trend has been observed in Golestan Province. In this series, intestinal-type tumours were the predominant type of adenocarcinomas, being twice as frequent as the diffuse-type tumours. This pattern and strong male predominance for gastric carcinomas in Golestan are similar to the patterns observed in other high-incidence rate areas of the world (Nomura, 1996).

ESCC was equally distributed between the two sexes, similar to the ratio in Linxian, but completely different from the pattern seen in Western countries, where the incidence of ESCC is low but is several-fold higher in men than in women (Munoz and Day, 1996).

The proportion of ESCC, EAC and GCA cases who were from rural areas or were of Turkmen ethnicity were similar and were higher than the proportions of these characteristics among GNCA or noncancer patients. Finding characteristics in which GCA patients are more similar to oesophageal cancer patients than to GNCA patients is also common in Linxian (Blot *et al*, 1993; Mark *et al*, 2000; Taylor *et al*, 2003).

Only one of the 144 ESCC cases had ever consumed alcohol, so alcohol cannot be considered a risk factor for ESCC in Golestan Province. It also appears that smoking is not a predominant risk factor. Only 27% of ESCC patients had ever smoked, and this proportion was similar to the proportion of smokers in the other cancer groups and noncancer patients. In addition, despite higher rates of smoking among men in Golestan (10.4% in men *vs* 0.4% in women in a large survey of the general population in this area (Noorbala and Mohammad, 1999)), ESCC incidence rates in this region are similar in men and women. A similar situation has been reported in Linxian, where smoking is much more common in men, the ESCC rates are similar in men and women, and the relative risk of ESCC associated with smoking is 1.6 (Guo *et al*, 1994; Abnet *et al*, 2001), making only 15% of the ESCC cases attributable to smoking. A totally different pattern is seen in Western countries: approximately 92% of all ESCC cases in the US have been attributed to smoking and alcohol consumption (Brown *et al*, 2001). However, it should be noted that most of the cases in most Western studies are men, and a recent study confined to women in the UK showed no association with alcohol and only a slight elevation of risk associated with smoking (Sharp *et al*, 2001).

Nass chewing, another locally suspected risk factor for ESCC, was seen in 12% of ESCC cases (18% of male and 6% of female patients), similar to the rates in the other cancer groups and in noncancer patients. The findings for the chewing of nass are in keeping with the results of the earlier case–control study which showed no elevation of risk for nass consumption (Cook-Mozaffari *et al*, 1979).

The consumption of opium, and in particular of opium dross (shireh and sukhteh), has been suspected to be a major aetiological factor for ESCC in Golestan Province (Joint Iran-IARC Study Group, 1977; Hewer *et al*, 1978; Malaveille *et al*, 1982; Dowlatshahi and Miller, 1985; Ghadirian *et al*, 1985). One-third (33%) of ESCC patients in our study had ever used opium, and similar proportions of opium usage were seen in the other patients. In order to evaluate the validity of such questionnaire responses concerning opium use among our patients, we recently measured opium metabolites in urines of 150 Golestan adults who responded to a similar questionnaire, evenly divided between those who admitted using opium and those who did not. Opium consumption reported by questionnaire was found to have a sensitivity and specificity of 90 and 91%, respectively, compared to urine results (Abnet *et al*., in press). So we think the questionnaire is reasonably valid. In the 1970s, however, almost half of the population tested from the high incidence areas had levels of morphine metabolites in the urine (Ghadirian *et al*, 1985; Munoz and Day, 1996), so there may have been a reduction of intake over the past 20 years. The role of opium and opium dross as aetiological factors for ESCC still needs further in-depth epidemiological investigation in this region, as does the role of hot tea, for which the earlier results were suggestive but equivocal (Cook-Mozaffari *et al*, 1979; Ghadirian, 1987). Hot drinks and stews have since been associated with elevated risk in other parts of the world (Munoz and Day, 1996).

The studies that were conducted in the 1970s in the Caspian Littoral and Golestan Province showed that areas inhabited by Turkmens had a much higher incidence rate of EC than areas with mainly Persian population, although the differences were less marked within Golestan Province (Kmet and Mahboubi, 1972; Mahboubi *et al*, 1973). In our study, approximately 50% of ESCC cases and other cancers were Turkmen patients. Based on the reports from the Iranian Ministry of Health, 51% of the population in Gonbad, Minoodasht and Kalaleh, the major city and towns referring patients to Atrak Clinic, are Turkmens. We are not sure that the percentage of Turkmen and non-Turkmen cancer patients referred to our clinic is the same, but if our ESCC patients are representative of all the ESCC patients in the area, then the risk of ESCC in Turkmens and non-Turkmens in this area is equal. If 70% of the incident cases of oesophageal cancer in eastern part of Golestan Province have been referred to the Atrak Clinic (as indicated by the interim results of the cancer registry) and if all the patients who have not been referred to our clinic were Turkmens, the risk of ESCC in Turkmens would be 1.99 that of the risk in non-Turkmens. This analysis argues against the existence of strong Turkmen-specific genetic components in the aetiology of ESCC in eastern Golestan. Only two (1%) of the ESCC cases were younger than 30 years of age and eight (4%) were younger than 40 years of age, another finding that argues against genetic factors being the predominant aetiological factors.

In summary, the results of this study show that ESCC is still the predominant type of upper GI cancer in high-risk areas of Golestan Province, and that tobacco, nass, alcohol and perhaps opium consumption are not the major aetiological factors for ESCC in this area. ESCC seems to be distributed among both Turkmens and non-Turkmens, and among both city and village dwellers. Therefore, it is likely that the main aetiologic factors for high incidence of ESCC are factors that are shared by most inhabitants of this area. Further case–control and cohort studies are needed to explore the risk factors associated in this region with the very high rates of ESCC.
